# Family functioning, psychological distress and suicidal tendencies among adolescents in Ghana

**DOI:** 10.4102/sajpsychiatry.v31i0.2382

**Published:** 2025-06-19

**Authors:** Mawuko Setordzi, Samuel Adjorlolo

**Affiliations:** 1 Department of Mental Health, College of Health Sciences, University of Ghana, Legon, Accra, Ghana; 2 Department of Nursing, Presbyterian Nursing, and Midwifery Training College, Dormaa Ahenkro, Ghana; 3 Research and Grant Institute of Ghana, Legon, Greater Accra Region, Ghana

**Keywords:** adolescents, family problem-solving, family communication, family affective involvement

## Abstract

**Background:**

Adolescence is characterised by profound changes in physical, cognitive, emotional and social functioning and is associated with psychological distress and suicidal tendencies. The family plays a significant role in safeguarding and promoting adolescents’ mental health. However, little is known about the influence of family functioning on adolescents’ psychological distress and suicidal tendencies in Ghana.

**Aim:**

This study examined the association between family functioning, psychological distress and suicidal tendencies among adolescents in Ghana.

**Setting:**

The study was conducted at three public senior high schools in Accra, Ghana.

**Methods:**

This cross-sectional study randomly recruited 600 students. We analysed the data using Spearman’s rho correlation and simultaneous multiple logistic regression.

**Results:**

The study revealed that poor family problem-solving had no statistically significant relationship with adolescents’ psychological distress and suicidal tendencies. After covarying by gender and age, poor family communication was not statistically and significantly associated with adolescent psychological distress but had a statistically significant positive association with suicidal tendencies. However, and more importantly, poor family affective involvement predicted both adolescents’ psychological distress and suicidal tendencies.

**Conclusion:**

These findings suggest the key role of the family in adolescents’ psychological distress and suicidal tendencies, emphasising the need for tailored family psychological interventions in Ghana.

**Contribution:**

This study contributes to the emerging literature on family functioning and its association with adolescent psychological distress and suicidal tendencies, underpinned by the McMaster model of family functioning (MMFF) in the Ghanaian setting.

## Introduction

The American Psychological Association (APA)^1^ defines psychological distress as:

[*A*] set of painful mental and physical symptoms that are associated with normal fluctuations of mood in most people. It is thought to be what is assessed by many putative self-report measures of depression and anxiety.

Adolescent psychological distress continues to be a significant global health concern. Despite the various national and international policy programmes that have been established to address this situation, there is still a cause for concern, particularly for low-middle-income countries (LMICs),^2,3,4^ primarily because of low prioritisation and underfunding of mental health interventions, research and practice.^5,6^ The mental health treatment gap exceeds 50% globally and about 90% in many LMICs, including Ghana,^7,8^ largely because of the limited mental health facilities and professionals in LMICs,^9^ thus affecting patients’ quality of life and lifespan.^10^ Adolescents’ psychological problems account for about 16% of the global disease burden and injury (measured by DALYs) among the world’s 1.2 billion adolescents (aged 10–19 years).^11^ Adolescence confers unique vulnerability to psychological problems because of the ontogenetic changes,^12^ easy access to and use of psychoactive drugs.^13^ Adolescents in this study are people aged 14–19, as stipulated by the World Health Organization (WHO).^14^

Globally, 10% – 20% of children and adolescents experience psychological disorders.^15,16^ In sub-Saharan Africa, where youths and young adults constitute 30% – 35% of the population,^17^ 14% reportedly suffer from psychological distress.^18^ In a related study, one in seven adolescents (14.3%) experiences significant psychological challenges, whereas one in ten (9.5%) qualifies for a psychiatric diagnosis.^19^

Relating specifically to Ghana, a sub-Saharan African (SSA) country, about 62% of Ghanaian youths were reported to have moderate to high common mental illnesses, including depression and anxiety.^20^ Although data on suicidal behaviour among adolescents are limited in Ghana, the media^5^ and survey reports^21^ suggest that suicidal behaviours are widespread, with the prevalence of suicidal ideation, suicidal plans and suicidal attempts estimated at 18.2%, 22.5% and 22.2%, respectively. These percentages vastly contrast with the 10.3% – 12.5% prevalence rates reported in high-income countries.^22,23^

The burden associated with psychological distress and suicidal tendencies is enormous, including stigmatisation and discrimination,^24^ household financial burden^25^ national economic loss^26^ and morbidity and mortality.^27^ For instance, the global cost involved in the treatment of psychological disorders in 2010 was estimated at 2.5 trillion US$, which is expected to rise to an estimated 6.0 trillion US$ by 2030.^28^ Relatedly, the healthcare cost associated with suicidal and self-harm tendencies and loss of productivity owing to the time spent away from work was estimated at $93.5 billion/per annum.^29^ Given the burden of mental health problems among adolescents and the limited mental health services in LMICs, it is essential to explore alternative and/or complementary, cost-effective strategies to inform evidence-based intervention programming and decision-making to improve adolescent mental health and well-being.

The family system is critical in influencing the mental well-being of adolescents.^30^ An adolescent’s mental health development largely depends on the family functioning.^31^ Family functioning is defined as the social and structural elements of the overall family context. It includes interactions within the family, the family’s problem-solving ability, adaptability, control, role performance, communication quality, affective expression and affective involvement.^32^ Among these family dimensions, affective involvement impacts the emotional needs of its members more. These family dimensions can function as either protective or risk factors for adolescents’ psychological well-being.^33^ In the Ghanaian context, the family system is a readily accessible support network that plays an instrumental role in promoting adolescents’ psychological well-being.

It provides support, encouragement and resilience, and aids in identifying symptoms of mental health problems early and promptly.

Previous studies conducted in Ghana^34,35^ are limited in scope relative to this study, which employed a multiple-factor approach. These studies focused on the stress of home life and gender role socialisation, family cohesion and parental conflict and their influence on adolescent psychological distress.^34,35^ The essence of the holistic family study approach and multiple outcome variables lies in its robustness in assessing complex mental health problems compared compared to approaches focused on individual family dimensions.^36^ The limited studies conducted in Ghana^34,35^ that examined the influence of family functioning on adolescent psychological distress did not take advantage of the insights from the McMaster Model of Family Functioning (MMFF). The use of the MMFF provides an important reference point for developing family interventions in the management of adolescent psychological distress and suicidal tendencies in Ghana. This study, therefore, examined the association between family functioning, psychological distress and suicidal tendencies among adolescents in the Accra Metropolis in Ghana. The study objectives were to determine the relationship between poor family problem-solving, poor family communication, poor family affective involvement and adolescents’ psychological distress and suicidal tendencies.

## Research methods and design

### Study design

The study used a self-report approach and a cross-sectional design to collect data from the respondents.

### Study population and sampling

The study was carried out in three public senior high schools (SHSs) in Ghana. The country’s educational system follows a 6–3–3–4 structure (i.e. primary school lasts 6 years, junior high lasts 3 years, senior high lasts 3 years and university bachelor’s lasts 4 years). The SHS were selected from the Accra Metropolis in Ghana’s capital city, Accra. The following schools were selected for inclusion in the study: Achimota SHS, located in Achimota, is a category A SHS; Wesley Grammar SHS is a Category B school located in Dansoman, while Kinbu Senior High School/Technical (SHST) Category C school is located at Tudu. All the schools were mixed-sex schools where both girls and boys were enrolled. The respondents were boys and girls aged 14–19 years, either in the first, second or third years in the 3-year SHS in Ghana. The inclusion of these categories enabled the investigation of family involvement in adolescents’ psychological distress and suicidal tendencies as a function of the different sociodemographic backgrounds.

Data were collected using a multi-stage sampling. Firstly, a stratified random sampling method was used to stratify all the SHSs in the Accra Metropolis into categories A, B and C of Ghana’s already existing Education Service categorisation. Secondly, a simple random sampling, approach was used to select one SHS from each stratum. Thirdly, systematic random sampling was also employed at the school level to choose the various classrooms from which the required number of respondents was identified. The first classrooms were randomly selected by drawing from a list of all classrooms. The first selected classroom became the starting point, and subsequent classrooms were chosen at sampling intervals until the required number was reached. We contacted the students in their respective classrooms and invited them to partake in the study if they consented. The students were briefed on the study’s objectives and responsibilities, and ethical issues, such as confidentiality, anonymity and withdrawal from the study without suffering any consequences, were explained. These ethical issues were adhered to throughout the study.

### Eligibility criteria

Inclusion criteria: 1. Respondents aged 14–19 years either in the first, second or third years. 2. Willingness to participate in the study.

Exclusion criteria 1. Individuals with disabilities, which made it difficult for them to respond to the questionnaire, for example, visual and hearing impairments.

### Sample size calculation

The study employed Yamane’s simplified formula to determine the sample size for the study.^37^ The formula is given by *n* = *N* 1+(*e*)2, where *n* = required sample size, *N* = the population size, 1 = a constant and *e* = margin of error (0.05) or significant level. With an anticipated non-response rate pegged at 30%, the final size of 600 was calculated.

### Data collection

First-, second- and third-year students who met the inclusion criteria and consented to participate in the study were guided to sign the consent and assent forms. Parents gave consent for their children to participate in the study by completing the written parental consent form. Data collection commenced on 16 January 2020, and ended on 09 March 2020.

### Measures

Family Assessment Device (FAD)^32^ subscales were used to measure family functioning. The original scale consists of 60 items based on the six dimensions of the MMFF. This study used three dimensions or subscales of the MMFF. These were family problem-solving, family communication and family affective involvement. The three subscales have a total of 22 items, rated on a 4-point Likert scale ranging from ‘strongly agree’ (1) to ‘strongly disagreed’ (4). Sample items include ‘when someone in the family is angry, the other family members know why’ and ‘we the family members resolve most everyday problems in the house’. The scores for a subscale were calculated by adding all the scores under the subscale and dividing them by the total number of items answered. The subscale scores range from 1.00, reflecting a healthy family functioning, to 4.00, reflecting an unhealthy family functioning. A good family functioning score is the average of all individual scores. In this study, the subscale problem-solving with six items had a Cronbach’s alpha of 0.76, communication with nine items had a Cronbach’s alpha of 0.83 and affective involvement with seven items had a Cronbach’s alpha of 0.83.

Patient Health Questionnaire (PHQ-4),^38^ an ultra-brief screening with 4 items rated on a 4-point Likert scale, was adopted to measure anxiety and depression. The scale responses range from 0 (not at all) to 3 (nearly every day). A total score on the PHQ-4 is obtained by summing individual items, which range from 0 to 12. Higher scores indicate a greater risk for anxiety and depression. Using the pre-existing guide, respondents can be categorised as follows: no psychological distress (0–2), mild (3–5), moderate (6–8) and severe (9–12). In this study, the Cronbach’s alpha for the PHQ-4 scale is 0.67.

Suicidal Behaviour Questionnaire-Revised (SBQ-R)^39^ scale was used to screen respondents for suicidal tendencies. Each of the 4 items on the SBQ-R taps into a distinct aspect of suicidality. Item 1 taps into lifetime suicidal thoughts and/or attempts. Item 2 examines how often suicidal thoughts have occurred over the last year. Item 3 measures the risk of suicide attempts. Item 4 evaluates the self-reported propensity for suicidal behaviour. The items were scored by summing the responses to the items. The SBQ-R total score ranges from 3 to 18. Higher scores indicate a greater risk for suicidal tendencies, with lower levels suggesting less risk. The Cronbach’s alpha for the SBQ-R scale is 0.80.

### Data analysis

Data were analysed with SPSS Version 23 (IBM Corp). Descriptive statistics were used to summarise the respondent’s sociodemographic characteristics. Inferential statistics, including Spearman’s rho correlation and simultaneous multiple logistic regression were conducted to address the study objectives. Spearman’s rho correlational analysis was performed to examine relationships between family functioning variables and adolescents’ psychological distress and suicidal tendencies. The outcome variables (i.e. adolescents’ psychological distress and suicidal tendencies) were dichotomised using cut-off points specified a priori by the respective scale developers. The variables poor family communication and poor affective involvement, which were significantly associated with the outcomes in bivariate analyses, were incorporated into the logistic regression model simultaneously and separately against psychological distress, stratified by the school categories.

The same analysis was repeated when the outcome variable was changed to suicidal tendencies. Simultaneous multiple logistic regression was used for regression analysis, having adjusted for gender and age as potential covariates. Simultaneous multiple logistic regression was used to estimate the influence of odds ratios of family functioning on adolescents’ psychological distress and suicidal tendencies. Psychological distress variables (anxiety and depression) and suicidal tendencies were coded 0 to reflect no/low risk and 1 to depict yes/high risk.

### Ethical considerations

Permission was obtained from the Greater Accra Regional Director of Education and the respective school management after presenting institutional ethics approval letters. Ethical clearance to conduct this study was obtained from the Noguchi Memorial Institute for Medical Research Institutional Review Board (No. NMIMR-IRB CPN 036/19-20), the Ghana Health Service Ethics Review Committee (No. GHS-ERC 033/12/19) and the Ghana Education Service (No. GES/GAR/HRMD/OL/2020/39), respectively. Respondents were contacted in their respective classrooms and invited to partake in the study. They were briefed about the study’s nature, scope, objectives and their rights and responsibilities. Ethical issues related to confidentiality, anonymity, voluntary participation and the right to withdraw from the study at any time without any form of consequences were explained to the respondents. Questionnaires were distributed to students who were willing and agreed to participate in the study by signing consent and assent forms.

## Results

### Demographic characteristics of respondents

A total of 600 respondents, that is, senior high school students from Achimota SHS, Wesley Grammar SHS and Kinbu SHS/T, were recruited for the study. The total sample comprised 275 (45.8%) boys and 325 (54.2%) girls. The average age of the respondents was 17.03 years (*SD* = 1.18, range: 14–19). In terms of the distribution of gender based on the categories of schools, the following was noted: in Category A school, there were 95 (47.5%) boys and 105 (52.5%) girls; for Category B, there were 76 (38%) boys and 124 (62%) girls; and, lastly, for Category C, there were 104 (52%) boys and 96 (48%) girls. The average age of respondents in Category A was 17.18 (*SD* = 0.88; range: 14–19), 16.54 for Category B (*SD* = 1.27; range: 14–19), and lastly, for Category C, the average age was 17.36 (*SD* = 1.18; range: 14–19). These were observed concerning the respondent’s religion: the majority were Christians (514 [85.7%]), while 86 (14.3%) were other religions. The respondents’ demographic characteristics are summarised in Table 1.

**TABLE 1:** Descriptive summary of respondents.

Description	Category A (*N* = 200)		Category B (*N* = 200)		Category C (*N* = 200)		Total (*N* = 600)	
	*n*	%	*n*	%	*n*	%	*n*	%
**Gender**								
Male	95.00	47.5	76.00	38.0	104.00	52.0	275.00	45.8
Female	105.00	52.5	124.00	62.0	96.00	48.0	325.00	54.2
**Age**								
M	17.18	-	16.54	-	17.36	-	17.03	-
s.d.	0.88	-	1.27	-	1.18	-	1.18	-
Minimum	14.00	-	14.00	-	14.00	-	14.00	-
Maximum	19.00	-	19.00	-	19.00	-	19.00	-
**Religion**								
Christians	181.00	90.5	177.00	88.5	156.00	78.0	514.00	85.7
Other Religions	19.00	9.5	23.00	11.5	44.00	22.0	86.00	14.3

M, mean; s.d., standard deviation.

### Bivariate relationship among the study variables

The result of the bivariate Spearman rho correlation was summarised in Table 2. It was found that family problem-solving had no statistically significant relationship with adolescents’ psychological distress, *r*(598) = 0.05, *p* < 0.25. Also, the relationship between family problem-solving and suicidal tendencies was not statistically significant, *r*(598) = 0.06, *p* < 0.12.

**TABLE 2:** Bivariate spearman rho correlation among the study variables.

Variables	1	2	3	4	5
1. Problem-solving	0.00	-	-	-	-
2. Communication	0.31**	0.00	-	-	-
3. Affective involvement	0.14**	0.23**	0.00	-	-
4. Psychological distress	0.05	0.11**	0.12**	0.00	-
5. Suicidal tendencies	0.06	0.11**	0.11**	0.30**	0.00

Note: 1. Problem-solving; 2. Communication; 3. Affective involvement; 4. Psychological distress; 5. Suicidal tendencies.

** , Correlation is significant at the 0.01 level (2-tailed).

### Predictors of adolescent psychological distress and suicidal tendencies

Two sets of simultaneous multiple logistic regression were used to examine the influence of family communication and affective involvement on adolescents’ psychological distress and suicidal tendencies. The simultaneous multiple logistic regression was conducted separately for the various categories of schools and the total sample. In all the analyses, gender and age were controlled as potential covariates. These variables were put in model 1, whereas the predictor variables (family communication and family affective involvement) were added to model 2. The family problem-solving scale was excluded from further analyses because of its lack of significant correlation with outcome variables in bivariate analysis (see Table 2).

The result of the multiple logistic regression is summarised in Table 3 and Table 4, respectively. Concerning adolescent psychological distress, it was observed that family communication was not a significant predictor among respondents in any of the categories of schools, as well as the total sample (AOR = 1.02, 95% CI = 1.00–1.05, *p* < 0.09). Family affective involvement emerged as a significant predictor of adolescents’ psychological distress for respondents in Category A (AOR = 1.07, 95% CI = 1.01–1.14, *p* < 0.03) and the total sample (AOR = 1.05, 95% CI = 1.02–1.09, *p* < 0.04) but not for Categories B and C. The odds of being classified as having psychological distress are high for respondents in Category A by 1.07, while in the total sample, the odds were 1.05 for a unit increase in poor family affective involvement.

**TABLE 3:** Simultaneous multiple logistic regression for predictor variables (family communication and family affective involvement) and adolescent psychological distress.

Predictor variables	Category A				Category B				Category C				Total Sample			
	B	Exp(B)	95% CI for Exp(B)		B	Exp(B)	95% CI for Exp(B)		B	Exp(B)	95% CI for Exp(B)		B	Exp(B)	95% CI for Exp(B)	
			Lower	Upper			Lower	Upper			Lower	Upper			Lower	Upper
**Model 1**																
Gender	−0.46	0.64	0.35	1.14	−0.02	−0.98	−0.54	1.79	−0.57	0.56	0.30	1.07	−0.33	0.72	0.51	1.01
Age	0.06	1.06	0.76	1.50	0.18	1.20	0.95	1.51	0.08	1.08	0.83	1.42	0.14	1.15	0.99	1.33
**Model 2**																
Communication	0.01	1.01	0.95	1.06	0.04	1.04	0.99	1.09	0.02	1.02	0.97	1.06	0.02	1.02	1.00	1.05
Affective involvement	0.07*	1.07	1.01	1.14	0.03	1.03	0.97	1.09	0.05	1.05	0.99	1.11	0.05*	1.05	1.02	1.09

Note: Predictor variables: Communication, Affective involvement. Outcome variable: Adolescent’s psychological distress.

CI, confidence interval; Exp, exponentiated regression coefficient (odds ratio).

* , *p* < 0.05. Analysis adjusted for gender and age.

**TABLE 4:** Simultaneous multiple logistic regression for predictor variables (communication and affective involvement) and adolescent suicidal tendencies.

Predictor variables	Category A				Category B				Category C				Total Sample			
	B	Exp (B)	95% CI for EXP (B)		B	Exp (B)	95% CI for EXP (B)		B	Exp (B)	95% CI for EXP (B)		B	Exp (B)	95% CI for EXP (B)	
			Lower	Upper			Lower	Upper			Lower	Upper			Lower	Upper
**Model 1**																
Gender	−0.30	0.74	0.39	1.40	0.10	1.11	0.56	2.21	−0.62	0.54	0.26	1.11	−0.29	0.75	0.51	1.10
Age	−0.08	0.93	0.64	1.34	0.05	1.05	0.80	1.37	0.03	1.03	0.77	1.38	0.01	1.01	0.86	1.19
**Model 2**																
Communication	0.01	1.01	1.07	0.96	0.07*	1.07	1.02	1.13	0.03	1.03	0.98	1.08	0.04*	1.04	1.01	1.07
Affective involvement	0.04	1.04	0.97	1.11	0.08*	1.08	1.01	1.16	0.05	1.05	0.98	1.12	0.05*	1.05	1.01	1.09

Note: Predictor variables: Communication, Affective involvement. Outcome variable: Suicidal tendencies.

CI, confidence interval; Exp, exponentiated regression coefficient (odds ratio).

* , *p* < 0.05. Analysis adjusted for gender and age.

Similarly, as shown in Table 4, family communication was a statistically significant predictor of suicidal tendencies for respondents in Category B schools (AOR = 1.07, 95% CI = 1.02–1.13, *p* < 0.01) and the total sample (AOR = 1.04, 95% CI = 1.01–1.07, *p* < 0.01). This means that for respondents in Category B schools, there were 1.07 odds of being classified as at-risk for suicidal tendencies, while in the total sample, the odds were 1.04 for every 1 unit increase in poor family communication. Likewise, family affective involvement was a significant predictor of suicidal tendencies among respondents in Category B (AOR = 1.08, 95% CI = 1.01–1.16, *p* < 0.03) and the total sample (AOR = 1.05, 95% CI = 1.01–1.09, *p* < 0.01). A unit increase in poor family affective involvement increases the risk of suicidal tendencies by 1.08 odds among respondents in Category B, while in the total sample, the odds were 1.05.

## Discussion

This is the first study in Ghana underpinned by the MMFF that examined the relationship between family functioning, specifically, poor family problem-solving, poor family communication, poor family affective involvement and adolescents’ psychological distress and suicidal tendencies. The results indicated no statistically significant relationship between poor family problem-solving and adolescents’ psychological distress, as well as suicidal tendencies.

Firstly, consistent with a previous study in Sweden,^40^ the current study found that poor family problem-solving was not significantly associated with adolescents’ psychological distress. However, the current study finding contradicts the bulk of prior studies^41,42^ that demonstrated a significant association between poor family problem-solving and adolescent psychological distress. Secondly, with respect to suicidal tendencies, the result corroborates a previous study in Iran^43^ that did not show any statistically significant relationship between poor family problem-solving and adolescent suicidal tendencies. However, it contradicts a previous study,^41^ which demonstrated that conflicts in the family, potentially reflective of poor family problem-solving, are associated with adolescents’ suicidal ideation and behaviour.

Plausibly, although developmental researchers have maintained that family relationships are crucial throughout adolescence,^44^ the peer relationship formed during adolescence may help adolescents quickly mobilise the social support created by their peers to mitigate problems in the family environment. Aside from the sociocultural nuances,^45^ which could explain the differing results, adolescent family conflict sometimes can promote resilience and positive psychological growth,^46^ thereby enhancing adolescents’ adaptive capacities. Therefore, the nature and effect of poor problem-solving in the family may have a negligible impact on psychological well-being and suicidal tendencies of these adolescents.

This resilience and positive psychological growth of adolescents is similar to the findings of,^34^ which indicated that resilience is a partial mediator of the relationship between stress and symptoms of anxiety and depression among 533 Ghanaian adolescents. The multifactorial explanations of suicidality could account for the differences in the results.^47^ Additionally, the nuanced meaning of suicidal thoughts and normative views of suicide in various religio-cultural contexts may explain the discrepancy in the findings. Further and importantly, suicidal behaviours are highly contextual, the reason for which suicidologists have recently recommended that context should be critically considered when evaluating the risk and protective factors of suicidality.^48^

Again, the result of the current study showed that poor family communication did not predict psychological distress among adolescents, which contradicts the literature on family communication and adolescents’ psychological distress in a previous study.^49^ Nevertheless, the current study’s finding corroborates a previous study in Iran.^50^ This finding may be partly explained by the presence of extra-familial relationships during adolescence, which could provide an avenue for alternative communication^51^ to buffer any faulty family communication that could provoke their psychological distress. Sociodemographic characteristics, including culture, may affect communication within the family. Therefore, cultural influences on family communication must be given much attention.

However, the current study revealed that poor family communication was a statistically significant predictor of suicidal tendencies among the respondents. This finding finds an explanation in the fact that when family communication is poor, adolescents may suppress their internal feelings and emotions, which is typical for children and adolescents.^52^ Worthy of note, concealing, deferring and/or dysregulating emotions negatively affect adolescents emotional well-being, leading to health-related consequences, such as anxiety and mood disorders.

Although this study did not reveal that poor family communication predicts adolescents’ psychological distress, a previous study found that poor family communication predicted adolescents’ psychological distress.^53^ Adolescent psychological distress has been projected as a major precursor to suicidal tendencies.^54^ It is, therefore, possible that one of the mechanisms contributing to the influence of suicidal tendencies is psychological distress, which suggests an indirect mechanism. Likewise, it also raises the possibility that family communication can directly influence suicidal tendencies without necessarily passing through psychological distress, suggesting a direct mechanism. This finding highlights the critical role of family-based counselling and psychoeducation programmes aimed at reducing adolescents’ suicidal tendencies by fostering a supportive family environment.

A previous study indicated that when adolescents have difficulty with effective communication with their parents, it results in an emotional impact that is akin to having no relationship at all.^55^ This increases adolescents’ emotional and psychological burden, which could potentiate an increase in their risk of suicidal tendencies. This finding is in tandem with the assertion of^56^ on his stress-vulnerability model and the development of the suicidal process that suggested that suicidality stems from the interaction between cognitive, affective and communication factors. Given the above, it is reasonable to conclude that ineffective communication is a risk factor for adolescents’ suicidal tendencies.

The present study supports an existing study^57^ that revealed that adolescent suicidality was associated with communication difficulties with parents. Even though this previous study was carried out in a different locale, the result demonstrated that poor family communication was associated with adolescent suicidal tendencies. Together, the current and previous findings underscore that suicidal tendencies transcend one’s geographical location, cultural variations and boundaries when family communication is particularly poor. This finding highlights the need for mental health policies and programmes to incorporate family-focused education, counselling and community-based initiatives to strengthen family–adolescent dialogue and emotional support. Additionally, this finding is a clarion call to policymakers to develop culturally sensitive strategies tailored towards addressing family dysfunction and its impact on adolescent suicidal tendencies.

The results from the current study revealed poor family affective involvement as a significant predictor of psychological distress and suicidal tendencies among the respondents in the current study. However, it is important to note that family cohesion between parents and adolescents becomes increasingly challenged during adolescence, as adolescents attempt to have behavioural and emotional autonomy from parents.^58^ This conflictual relationship may worsen any existing problematic family affective involvement and further exacerbate the family environmental stress. This stress increases the risk of psychological distress and suicidal tendencies, highlighting a possible reason for the current study finding. Regarding psychological distress, the current study is in keeping with the findings of a previous study,^59^ which found that a poor parent–adolescent relationship is associated with an increased risk of depressive symptoms.

More so, concerning adolescent suicidal tendencies, the current finding concurs with a prior study,^60^ which demonstrated that poor family affective involvement is associated with adolescent suicidal tendencies. There is a general consistency between prior and current studies, revealing a significant relationship between poor family relationships and adolescents’ psychological distress and suicidal tendencies. A possible social factor could explain this consistency across different countries. The unique roles of social variables have been postulated in extant psychological and sociological theories of suicide,^61^ which often culminates in suicidal tendencies.

## Limitations

The use of a cross-sectional design limits the extent to which causality could be inferred. A longitudinal design would have been more helpful to infer this causality. The sensitive nature of the study could have led to social desirability responses because of the health-related stigma that surrounds these disorders: anxiety, depression and suicidal tendencies. Again, one methodological shortfall included the use of adolescents’ self-report responses, which could have also led to either underreporting or over-reporting of family functioning and symptoms of anxiety, depression and suicidal tendencies. Another pitfall of the study was the use of an average population across the three categories of schools. Ideally, a proportionate sampling equivalent to each school population could have been more appropriate and representative. Also, not all aspects of the family function were studied, given the scope of this study. Given the above, caution should be taken when generalising this study’s results. These limitations notwithstanding, this study adds to the limited literature addressing family functioning and adolescents’ psychological distress and suicidal tendencies, especially in Ghana.

## Conclusion

This study has found that poor family problem-solving did not significantly correlate with both adolescents’ psychological distress and suicidal tendencies. Also, poor family communication was not significantly associated with adolescents’ psychological distress but with suicidal tendencies. Notably, poor family affective involvement predicted both adolescents’ psychological distress and suicidal tendencies. Because of many family dimensions and dynamics and the unique strength of the family, there is a need to look at multiple family factors and their collective influence on adolescents’ psychological distress and suicidal tendencies. A holistic approach to family functioning not only safeguards adolescents’ but also contributes to saving the scarce resources of the country in managing these psychological disorders.
